# Complete mitochondrial DNA sequence of yellow-legged buttonquail (*Turnix tanki*)

**DOI:** 10.1080/23802359.2021.1935341

**Published:** 2021-06-07

**Authors:** Xue Gou, Shize Li, Cheng Wang, Han Fan, Caichun Peng, Canshi Hu, Mingming Zhang, Haijun Su

**Affiliations:** a Forestry College, Guizhou University, Guiyang, China; b Research Center for Biodiversity and Natural Conservation, Guizhou University, Guiyang, China; c College of Life Sciences, Guizhou University, Guiyang, China; d Department of Food Science and Engineering, Moutai Institute, Renhuai, China

**Keywords:** Complete mitogenome, gene arrangement, Yellow-legged Buttonquail, mitochondrial DNA, *Turnix tanki*

## Abstract

The Yellow-legged Buttonquail *Turnix tanki* is a species of the genus *Turnix*, which belongs to the order Charadriiformes. It is distributed across almost all of China. The International Union for Conservation of Nature has assessed the bird’s conservation status as ‘Least Concern (LC).’ We sequenced the complete mitogenome of *T. tanki* and examined its phylogenetic relationship with other charadriiformes species. The mitochondrial DNA is packaged in a compact 17,620 base pair circular molecule with A + T content of 57.90%. It contains 37 typical mitochondrial genes, including 13 protein-coding genes, two rRNAs and 22 tRNAs, and two non-coding regions. We reconstructed a phylogenetic tree based on mitogenome sequences of five Turnicidae species and one outgroup. Phylogenetic analysis indicated that *T. tanki* is a sister to *T. suscitator.*

The Yellow-legged Buttonquail *Turnix tanki* is a species of the genus *Turnix*, which belongs to the order Charadriiformes. It is reported to have a very large and stable range over 20,000 km^2^, which places it under the threshold for vulnerable status. It is listed as the Least Concern (LC) species by International Union for Conservation of Nature and Natural Resources (IUCN, 2016), supported by the estimated population, which is not believed to approach the thresholds vulnerable status (<10,000 mature individuals with a continuing decline estimated to be >10% in 10 years or three generations (BirdLife International 2020)). It has also been listed as a LC species on the Red List of China’s Vertebrates (Jiang et al. 2016). They often build their nests in the grass on the ground or in wheat and soybean fields, usually using the shallow pit to build the nest, and covered by the surrounding crops or weeds. The Yellow-legged Buttonquail looks like a quail, but it is smaller. Its back, shoulders, waist, and tail are grayish brown with small black and brown spots. The tail is short and the central tail feather is not elongated. The Yellow-legged Buttonquail is not good at singing, but it runs fast on the ground. It usually runs away from danger rather than taking to the air. It mainly feeds on plant buds, berries, grass seeds, grains, and insects and other small invertebrates (Zhao, 2001). Here, we report the complete mitogenome of *T. tanki* (GenBank number: MW307919) and examined its phylogenetic relationship with other Charadriiformes species whose mtDNA data are available.

The specimen (NO. GZUNZ20201129003; contact person: Xue Gou; email: xuemiao2@hotmail.com) was collected from Huaxi in the Guiyang on 21 November 2019 (E106°40′19.60″, N26°25′58.42″), and was stored in zoological specimens room of Research Center for Biodiversity and Nature Conservation of Guizhou University. Unilateral legs were taken, exoskeleton was removed carefully, and then muscle tissue was gathered for mtDNA isolation. The mitochondrial genome of Yellow-legged Buttonquail was extracted by the Mitochondrial Extraction Kit (Beijing Aidlab Biotechnologies Co., Ltd.). The mitochondrial genomes of *T. velox* (MN356355.1) were used to design primers for polymerase chain reaction (PCR) and used as a template for gene annotation.

The complete mtDNA sequence of the Yellow-legged Buttonquail is 17,620 bp in length. Its overall base composition is A, 31.40%; C, 29.80%; G, 12.30% and T, 26.50%. The A + T content is 57.90%. It has a typical circular mitochondrial genome containing 13 protein-coding genes, 22 transfer RNAs, two ribosomal RNAs, and two non-coding A + T-rich regions, which is typical in birds (Boore 1999). The order and orientation are identical to the standard avian gene order (Gibb et al. 2007). Of the 13 protein-coding genes, 11 utilize the standard mitochondrial start codon ATG. However COI uses GTG and ND3 uses ATT as the initiation codon. TAA is the most frequent stop codon, although COIII, ND3, and ND4 end with the single nucleotide T; COI, ND1, and ND6 end with AGG; and ND2 ends with TAG.

The 12S rRNA is 969 bp in length, and the 16S rRNA is 1590 bp in length, which are located between tRNA-Phe (gaa) and tRNA-Leu (taa), and separated by tRNA-Val (tac). All tRNAs have the classic cloverleaf secondary structure, as observed in other bird mitogenomes (Bernt et al. 2012). Most of the mitochondrial genes are encoded on the heavy strand (H-strand), but ND6 and eight tRNA genes are instead encoded on the light strand (L-strand) (Table 1).

**Table 1.** Organization of the complete mitochondrial genome of Yellow-legged Buttonquail *Turnix tanki*.

Gene	Position	Size	Spacer (+) or Overlap (–)	Codon		Anti-codon	Strand
	Start–End			Start	Stop		
tRNA-Phe	1–69	69				GAA	H
12S rRNA	70–1038	969					H
tRNA-Val	1039–1107	69				TAC	H
16S rRNA	1108–2697	1590					H
tRNA-Leu	2698–2771	74				TAA	H
ND1	2779–3756	978	7	ATG	AGG		H
tRNA-Ile	3755–3827	73	−2			GAT	H
tRNA-Gln	3855–3925	71	27			TTG	L
tRNA-Met	3925–3993	69	−1			CAT	H
ND2	3994–5034	1041		ATG	TAG		H
tRNA-Trp	5033–5102	70	−2			TCA	H
tRNA-Ala	5104–5172	69	1			TGC	L
tRNA-Asn	5176–5250	75	3			GTT	L
tRNA-Cys	5253–5319	67	2			GCA	L
tRNA-Tyr	5319–5389	71	−1			GTA	L
COI	5391–6941	1551	1	GTG	AGG		H
tRNA-Ser	6933–7006	74	−9			TGA	L
tRNA-Asp	7009–7077	69	2			GTC	H
COII	7079–7762	684	1	ATG	TAA		H
tRNA-Lys	7764–7832	69	1			TTT	H
ATP8	7834–8001	168	1	ATG	TAA		H
ATP6	7992–8675	684	−10	ATG	TAA		H
COIII	8675–9458	784	−1	ATG	T		H
tRNA-Gly	9459–9527	69				TCC	H
ND3	9528–9877	350	1	ATT	T		H
tRNA-Arg	9878–9946	69				TCG	H
ND4L	9948–10,244	297	1	ATG	TAA		H
ND4	10,238–11,615	1378	−7	ATG	T		H
tRNA-His	11,616–11,685	70				GTG	H
tRNA-Ser	11,686–11,751	66				GCT	H
tRNA-Leu	11,751–11,820	70	−1			TAG	H
ND5	11,821–13,641	1821		ATG	TAA		H
Cytb	13,649–14,791	1143	7	ATG	TAA		H
tRNA-Thr	14,796–14,865	70	4			TGT	H
D-LOOP	14,866–16,785	1920					
tRNA-Pro	16,786–16,855	70	1920			TGG	L
ND6	16,883–17,404	522	27	ATG	AGG		L
tRNA-Glu	17,408–17,481	74	3			TTC	L
D-LOOP	17,482–17,620	139					

We used MEGA6 to construct a phylogenetic tree using the maximum-likelihood method (Figure 1) based on the mitogenome sequences of *T. tanki* from the other five Turnicidae species and one outgroup according to a previous study. The results showed *T. tanki* to be a sister to *T. suscitator* (Figure 1).

**Figure 1.**
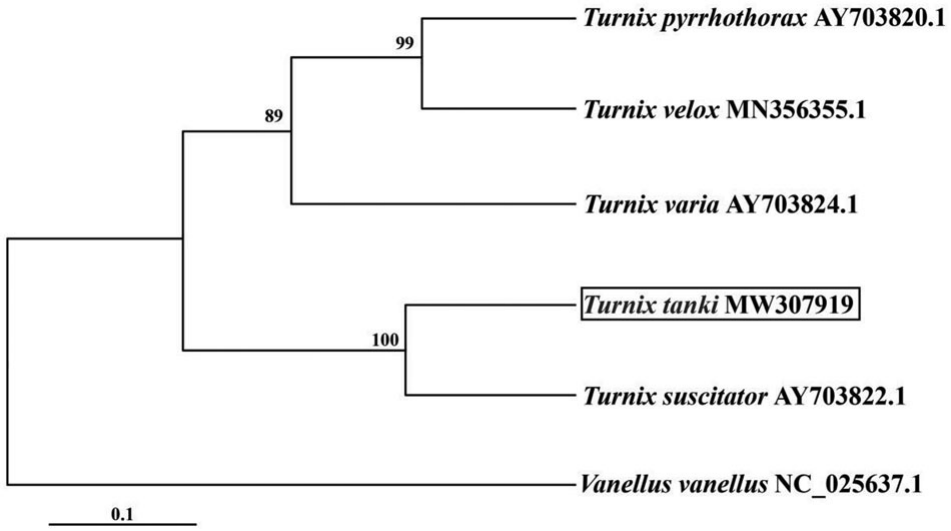
Maximum likelihood tree based on mitogenome sequences of five Turnicidae species and one outgroup. Bootstrap values were presented under the branch, and the posteriori probability values are omitted because all of them are 100%.

This study is the first to report and analyze the complete mitochondrial genome of Yellow-legged Buttonquail, *T. tanki*. The complete mitogenome of *T. tanki* may facilitate conservation of this non-flagship species and provide fundamental genetic data for research into the evolution of Turnicidae.

## Data Availability

Mitogenome data supporting this study are openly available in GenBank at: https://www.ncbi.nlm.nih.gov/nuccore/MW307919. Associated BioProject accession numbers are https://www.ncbi.nlm.nih.gov/bioproject/PRJNA724019.
